# Claudin-1 enhances tumor proliferation and metastasis by regulating cell anoikis in gastric cancer

**DOI:** 10.18632/oncotarget.2936

**Published:** 2014-12-02

**Authors:** Jie Huang, Li Zhang, Changyu He, Ying Qu, Jianfang Li, Jianian Zhang, Tao Du, Xuehua Chen, Yingyan Yu, Bingya Liu, Zhenggang Zhu

**Affiliations:** ^1^ Shanghai Key Laboratory of Gastric Neoplasms, Department of Surgery, Shanghai Institute of Digestive Surgery, Ruijin Hospital, Shanghai Jiao Tong University School of Medicine, Shanghai, China

**Keywords:** Claudin-1, Anoikis, β-catenin, Gastric cancer

## Abstract

Claudin-1 (CLDN1) is overexpressed in gastric cancer and correlated with tumor invasion, metastasis and poor outcome. Here, we both down and up regulated CLDN1 expression in gastric cancer cells to elucidate its role in gastric carcinogenesis and tumor progression. We found that deficiency of CLDN1 inhibited cells migration, invasion, and colony formation *in vitro* and tumorigenicity, metastasis *in vivo*. Also, CLDN1 promoted cell aggregation and increased anoikis resistance. Down or up regulation of CLDN1 was accompanied with changes of membrane β-catenin expression as well as Akt and Src activities. When β-catenin was up-regulated in CLDN1-KD cells, cell aggregation and anoikis resistance were restored, and Akt and Src signal pathways were re-activated. Taken together, these findings suggest that CLDN1 is oncogenic in gastric cancer and its malignant potential may be attributed in part to regulation of anoikis, by mediating membrane β-catenin-regulated cell-cell adhesion and cell survival.

## INTRODUCTION

Tight junctions (TJs) are the important functional constitutes in cell-cell adhesion of normal epithelium, they mechanically link cells, form the epithelial barrier against paracellular transport and maintain epithelial cell polarity [1, 2]. As the most important structural and functional components of TJs, claudins (CLDNs) have been found involved in various pathological conditions, including abnormal inﬂammation and tumor progression [3]. Twenty seven distinct CLDNs have been cloned in mammals. The expression of different subtypes of CLDNs has tissue-specific feature and is appeared to be regulated differentially in cancers of diverse tissue origin [4-6]. Dysregulation and remodeling of CLDNs may cause change of cell polarity and cell-cell adhesion structures which is associated with a transform in motility, invasiveness and metastasis in epithelial cancers [7].

Gastric cancer, the second leading cause of cancer death worldwide, the heterogeneous expression of CLDNs subtypes such as claudin-1, -3, -4, -6, -7, -9 and -18 have been found. CLDN3 and CLDN4, previously classiﬁed as intestinal claudin phenotypes, have an important role in tumor metastatic pathway [8]. Down-regulation of CLDN3 is associated with proliferative potential in early gastric cancers [9]. Membranous claudin-4 expression is associated with gastric cancer progression and prognosis in gastric carcinoma [10]. In addition, individual increased expression of CLDN6, CLDN7, or CLDN9 is sufficient to enhance migration, invasion and proliferation of gastric cancer cell [11]. Downregulation of CLDN18 expression, possibly regulated by PKC/MAPK/AP-1 pathway and DNA methylation, is associated with poor survival in gastric carcinoma [12-14].

For CLDN1, its overexpression in gastric cancer is related with tumor invasion and metastasis [15, 16]. We recently reported that CLDN1 was highly expressed in intestinal-type gastric cancer and correlated with advanced TNM stage, lymph node metastasis and poor outcome [17]. However, the role and detailed mechanism of CLDN1 in gastric cancer are still unknown. Previous findings suggested that CLDN1 was an EMT marker [18] and responsible for EMT related cell behaviors including migration, invasion, and matrix metalloproteinase (MMP) activation [19]. Earlier reports indicated that CLDN1 recruited and activated MMP-2 and MMP-9, which enhanced cell invasion and metastasis [20-22]. The dissent was that inhibition of CLDN1 in colon cancer cell induced a significant decrease only in MMP-9 activity, but not in MMP-2 activity, which suggested other mechanisms might be involved in the CLDN1 related invasion and metastasis.

Another interesting finding recently reported in colon cancer, was that CLDN1 protected cells from anoikis in suspension [23]. Anoikis, a special form of apoptosis occurring when cells detach from the extracellular matrix (ECM), is a key mechanism in maintaining tissue homeostasis and development [24]. Failure to execute the anoikis program may result in cells surviving under suspension conditions or proliferating at ectopic sites, this deregulation of anoikis execution is considered as an emerging hallmark of cancer cells and contributes to the formation of metastasis in distant organs [25, 26]. Claudin-1 regulated cell anoikis in colon cancer in a manner of regulating E-cadherin expression, β-catenin/Tcf and Src signaling [23, 27]. Integrity of cell-cell contacts prevents anoikis involving survival signal pathways activation of β-catenin, Src and PI3-K/Akt [23, 28]. Indeed, there is a mutual interaction between CLDN1 and β-catenin [17, 27, 29]. The role of CLDN1 and β-catenin in the gastric tumorigenesis and metastasis piqued our interest in the further study.

In the current study, we demonstrated that knockdown of CLDN1 expression in gastric cancer cells inhibited tumor growth and metastasis *in vitro* and *in vivo* by inducing cell anoikis. Mechanically, we further discovered that CLDN1 enhanced cell anoikis resistance through mediating membrane β-catenin expression followed by inducing cells aggregation and inhibiting apoptosis cascade.

## RESULTS

### Knockdown of CLDN1 inhibits cell growth in soft agar 3D but not in monolayer culture condition

Given that CLDN1 is markedly up-regulated in gastric cancer tissus [17] and cell lines (Figure S1A-B), we hypothesized that CLDN1 may function as an oncogene. To elucidate the role of CLDN1 in gastric carcinogenesis, we constitutively knocked down CLDN1 expression in the human cancer cells BGC-823 and HS-746T using shRNA (BGC-823/CLDN1-KD and HS-746T/CLDN1-KD). Compared with negative controls, both mRNA and protein levels of CLDN1 were significantly down-regulated in BGC-823/CLDN1-KD and HS-746T/CLDN1-KD cells assayed by QRT-PCR (Figure S1C), WB (Figure S1D) and immunofluorescence staining (Figure S1E).

**Figure 1:**
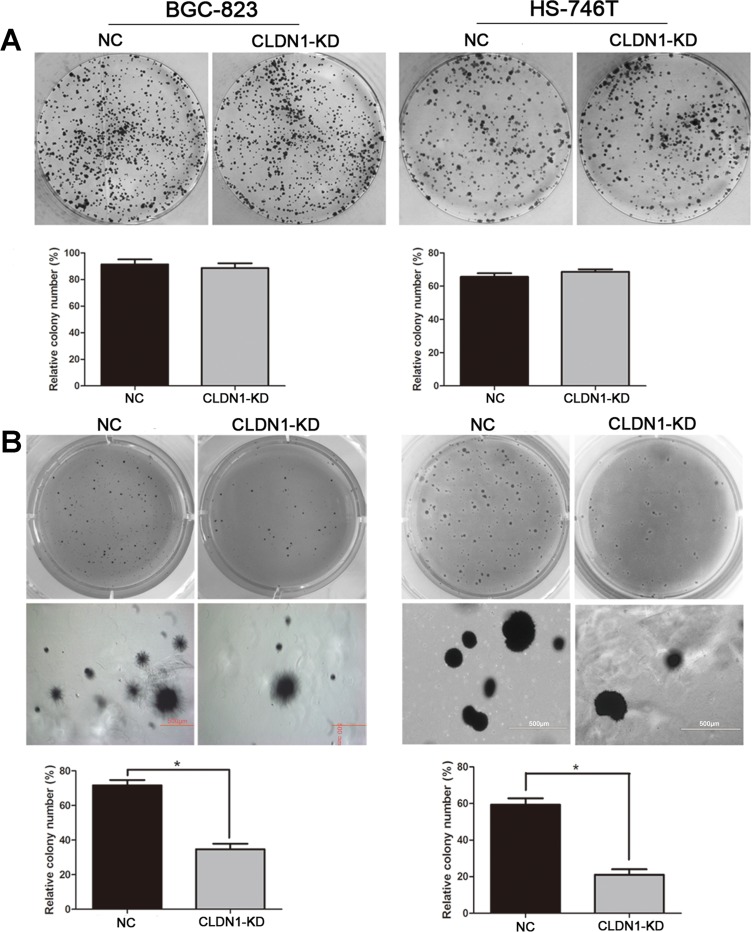
Knockdown of CLDN1 inhibits cell growth in soft agar 3D but not in monolayer culture condition (A) Plate colony formation assay of BGC-823/CLDN1-KD and HS-746T/CLDN1-KD and control cells on regular culture plates after 14 days of culture. Relatively colony numbers of CLDN1-KD cells were similar to that of control cells in relatively colony formation assay. (B) Soft agar colony formation assay of BGC-823/CLDN1-KD and HS-746T/CLDN1-KD and control cells in soft agar after 14 days of culture (under eye view and microscope of 40 magnifications). Relatively colony numbers of CLDN1-KD cells significantly decreased as compared to that of control cells (*, P < 0.05).

We firstly tested cell proliferation, apoptosis and cell cycle *in vitro*. As results, in monoplayer culture, CLDN1 did not affect cell growth (Figure S2A), the cell cycle distribution (Figure S2B) and apoptotic rate (Figure S2C). In the following colony formation assays, the colony numbers on culture plates were similar between CLDN1-KD groups and control groups (Figure 1A). However, when cultured in 3D soft agar, CLDN1-KD cells of BGC-823 and HS-746T exhibited fewer and smaller clonies compared with vector-control cells (Figure 1B). These data suggest that CLDN1 is critical in maintaining cell growth in 3D culture.

### Knockdown of CLDN1 suppresses cell migration and invasion *in vitro*

**Figure 2:**
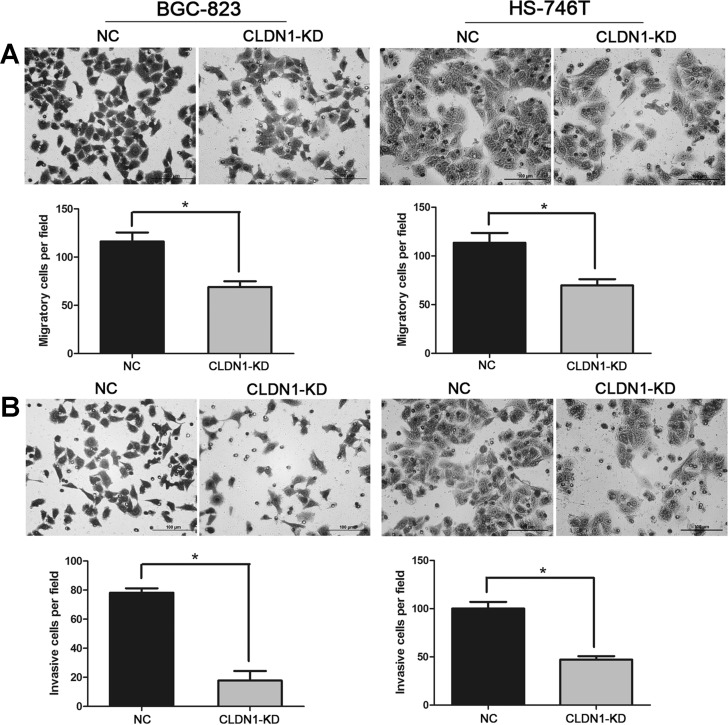
Knockdown of CLDN1 inhibits cell migration and invasion *in vitro* (A) Representative photographs of migratory cells on the membrane in cell migration assay (original magnifications: ×200). Average migratory cell number of CLDN1-KD cells were significantly lower than that of negative control cells (*, P < 0.05). (B) Representative photographs of invasive cells on the membrane in cell invasion assay (original magnifications: ×200). Average migratory cell number of CLDN1-KD cells were significantly lower than that of negative control cells (*, P < 0.05).

To explore the effect of CLDN1 on tumor metastasis, cell migration and invasion were tested by using a Boyden chamber assay. We found that CLDN1-KD significantly decreased the number of migrated cells (Figure 2A). Consistently, knockdown of CLDN1 in gastric cancer cells inhibited cell invasive ability (Figure 2B). These data suggest that knockdown of CLDN1 expression in gastric cancer cells suppresses metastasis.

### Knockdown of CLDN1 suppresses tumorigenesis and metastasis *in vivo*

**Figure 3:**
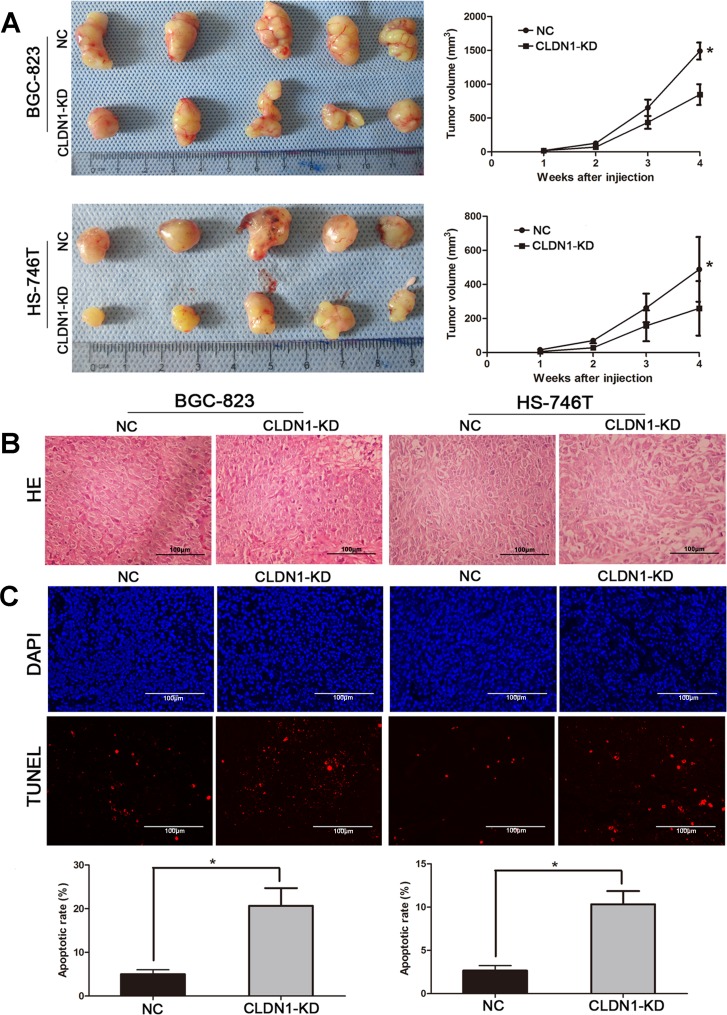
Knockdown of CLDN1 suppressed tumorigenesis and increased apoptosis *in vivo* (A) The photograph shows representative features of tumor xenografts 4 weeks after inoculation. Tumor growth curves were measured after injection, and tumor diameters were measured every 7 days. The values are given as mean ± SD (*, P < 0.05). (B) Representative images of H&E staining of tumor (original magnifications: ×200). (C) Representative images of TUNEL assay of tumor xenografts (original magnifications: ×200). Average apoptotic rate of five independent fields given as mean ± SD. (*, P < 0.05).

We further test whether CLDN1-KD could inhibit tumor growth and metastasis *in vivo*. To do so, we transplanted BGC-823/CLDN1-KD and HS-746T/CLDN1-KD cells into nude mice by subcutaneously and by tail vein, and transplanted BGC-823/NC and HS-746T/NC as control. We found that tumor growth was significantly suppressed in CLDN1-KD groups compared with control groups (Figure 3A). The average size of tumors formed by CLDN1-KD cells at day 28 was significantly smaller than those tumors formed by control cells (P < 0.05). Thus, knockdown of CLDN1 suppressed tumor tumorigenesis in mice. In addition, H&E staining of tumors showed that NC tumor cells arranged more closely than CLDN1-KD cells (Figure 3B). TUNEL analysis also showed more apoptotic cells in CLDN1-KD (Figure 3C). Collectively, these results indicated that CLDN1 played a role in gastric cancer tumorigenesis and metastasis.

**Figure 4:**
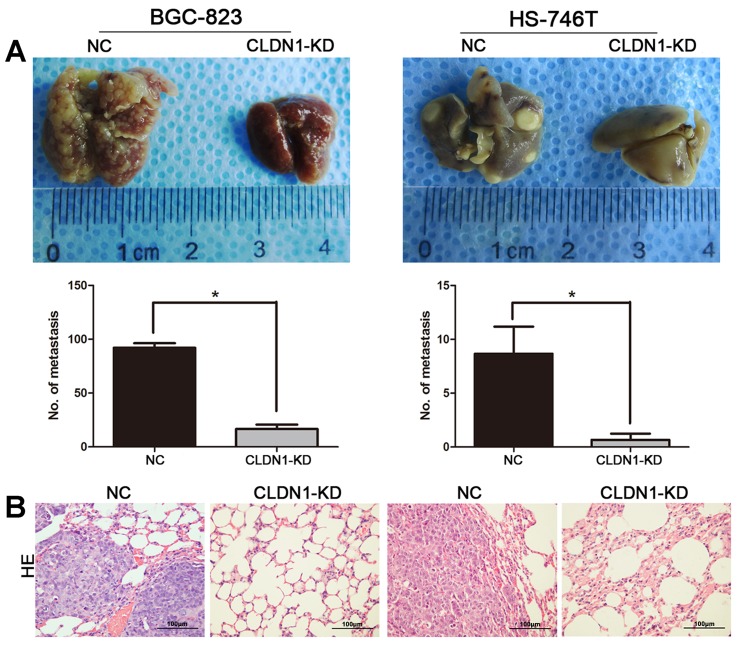
Knockdown of CLDN1 suppressed metastasis *in vivo* (A) Photographs of pulmonary metastases derived from the mice with tail vein injection. Average number of lung metastatic nodes per mouse were given as mean ± SD (*, P < 0.05). (B) Representative images of H&E staining of tumor xenografts (original magnifications: ×200).

Among the mice injected tumors through tail vein, knockdown of CLDN1 remarkably suppressed the formation of lung metastases under naked eye (Figure 4A). Quantitative data showed that the number of metastatic nodules formed by BGC-823/CLDN1-KD and HS-746T/CLDN1-KD cells were significantly fewer than that of BGC-823/NC and HS-746T/NC cells. The images of H&E staining of tumor xenografts were shown in Figure 4B.

### Knockdown of CLDN1 induces anoikis by activating caspase-3 pathway

**Figure 5:**
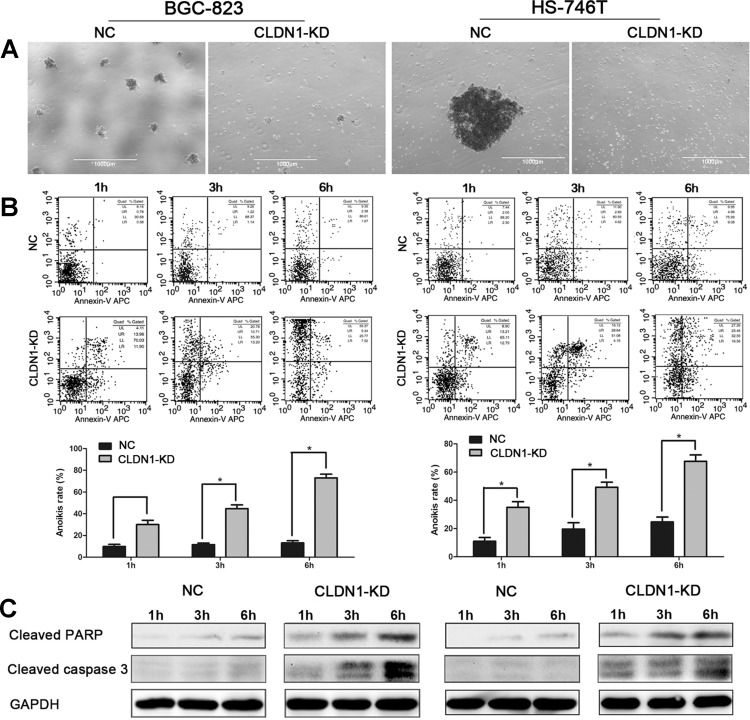
Knockdown of CLDN1 induces anoikis by activating caspase-3 pathway (A) Representative photographs of cell aggregation after 6 hrs of suspension (original magnifications: ×40). (B) Representative histograms depicting apoptosis and relative apoptotic rate of CLDN1-KD cells and their respective controls after 1h, 3h and 6h of suspension determined by FCM (*, P < 0.05). (C) Protein expression of cleaved caspase-3 and cleaved PARP in CLDN1-KD cells increased after 1h, 3h and 6h of suspension as compared to that of control cells by immunoblotting. These results were repeated in three independent experiments.

Previous data showed that CLDN1-KD defected cell growth in 3D *in vitro* culture and *in vivo* metastasis, suggesting that CLDN1 might be involved in anti-anoikis regulation. To further explore the possible role of CLDN1 in regulating anoikis, we employed an anoikis experimental model. The cells were plated on poly-HEMA coated 30 mm culture dishes and shaked at 80 rpm in the incubator. As result, we found that control cells formed large spheres in suspension culture. In contrast, CLDN1-KD cells lacked the ability to form spheres (Figure 5A). Apoptosis rate in control and CLDN1-KD cells cultured in suspension was then labeled by Annexin V/PI staining followed by FACS. As shown in Figure 5B, the apoptotic rates in CLDN1-KD cells were significantly higher than those in control cells at 1h, 3h and 6h cultured in suspension. As the key factor in apoptosis is the cleavage of caspase-3, we examined the levels of cleaved caspase-3 and cleaved PARP using cell lysates from suspension culture. As expected, we found that the levels of cleaved caspase-3 and cleaved PARP were dramatically increased in CLDN1-KD cells (Fig. 5C). These data suggest that knockdown of CLDN1 expression in gastric cancer cells induces anoikis with the activation of caspase-3 pathway.

### Overexpression of CLDN1 up-regulates cell migration, invasion and colony formation abilities and anoikis resistance

**Figure 6:**
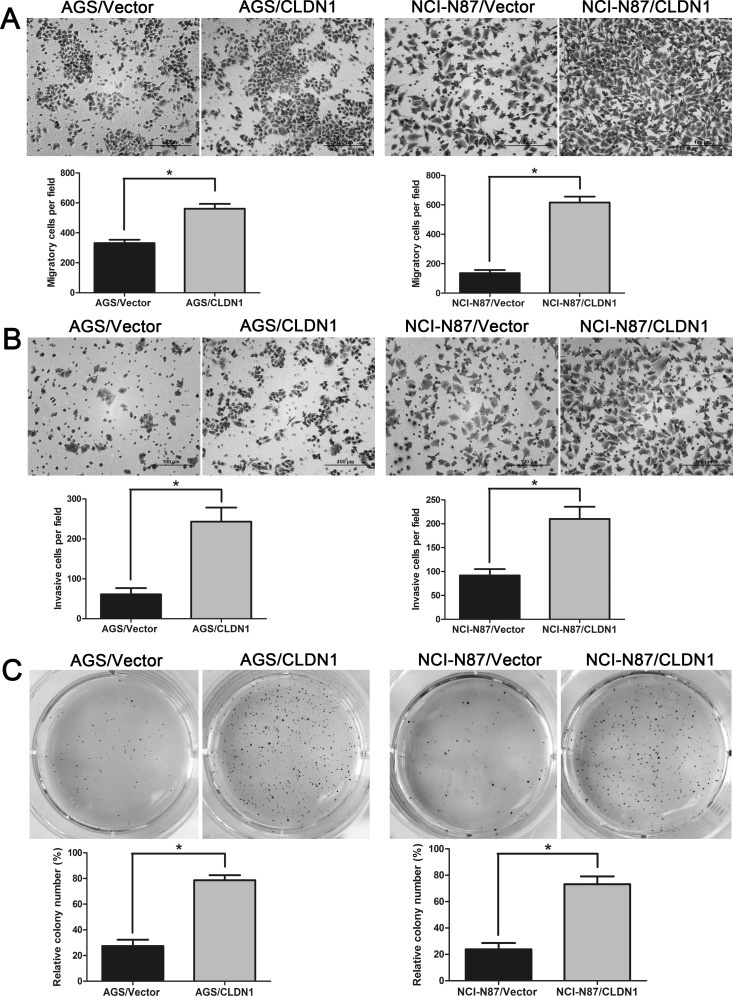
Overexpression of CLDN1 in gastric cancer cells AGS and NCI-N87 enhances cell migration and invasion *in vitro*, and facilitates cell growth in soft agar 3D (A) Representative photographs of migratory cells on the membrane in cell migration assay (original magnifications: ×200). Average migratory cell number of CLDN1-overexpressed cells were significantly higher than that of control cells (*, P < 0.05). (B) Representative photographs of invasive cells on the membrane in cell invasion assay (original magnifications: ×200). Average migratory cell number of CLDN1-overexpressed cells were significantly higher than that of control cells (*, P < 0.05). (C) Soft agar colony formation assay of CLDN1-overexpressed cells and control cells in soft agar after 14 days of culture under eye view. Relatively colony numbers of CLDN1-overexpressed cells significantly increased as compared to that of control cells (*, P < 0.05).

**Figure 7:**
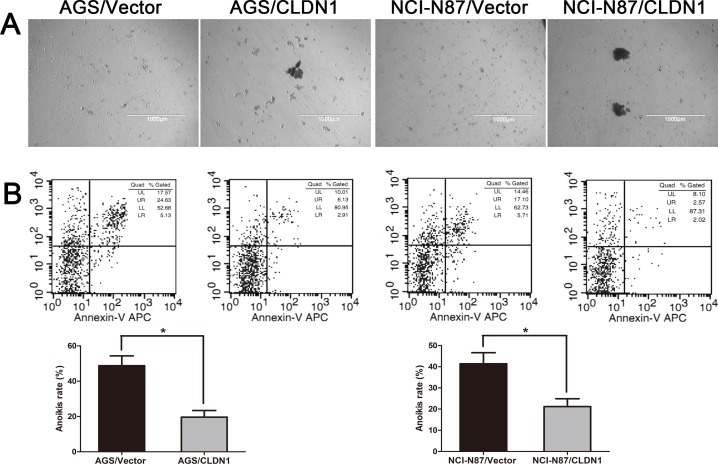
Overexpression of CLDN1 enhanced cell aggregation and anoikis resistance (A) Representative photographs of cell aggregation after 6 hrs of suspension (original magnifications: ×40). (B) Representative histograms depicting apoptosis and relative apoptotic rate of CLDN1-overexpressed cells and their respective controls after 3h of suspension determined by FCM (*, P < 0.05).

To reinforce its oncogenic functions, we up-regulated CLDN1 expression in two gastric cancer cell lines of AGS and NCI-N87. The effect of CLDN1 overexpression was verified by QRT-PCR (Figure S3A) and WB (Figure S3B). In the following cell functional tests, we found that overexpression of CLDN1 increased the numbers of migrated cells in the transwell migration and invasion assays (Figure 6A-B), promoted cell growth in 3D soft agar (Figure 6C) and cell aggregation in suspension (Figure 7A), and decreased cell anoikis (Figure 7B). These opposite performance to the CLDN1-KD cells reinforce that CLDN1 might initiate gastric cancer generation and metastasis by maintaining anoikis resistance.

### Membrane β-catenin expression as well as Src and Akt activities were involved in CLDN1 regulated anoikis

The above study showed CLDN1 affected cell aggregation in suspension culture, we hypothesized that cell-cell connection might be compromised. In addition, our previous finding showed that membrane β-catenin expression was positively correlated with CLDN1. β-catenin might partake in the process of CLDN1 regulated anoikis which had been mentioned in colon cancer.

**Figure 8:**
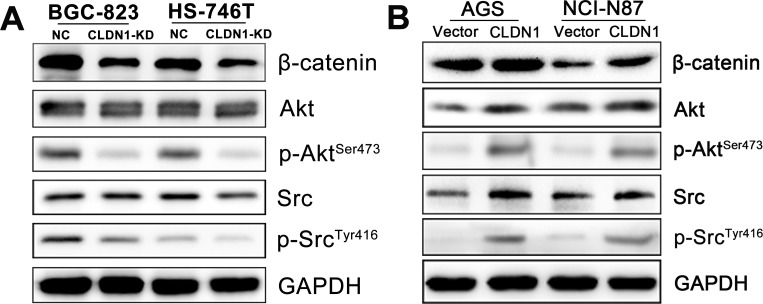
Involvement of β-catenin and survival signal of Akt and Src in the process of CLDN1 regulated anoikis (A) WB analysis, as compared to negative control cells, CLDN1-KD cells showed a distinct decrease of β-catenin, phosphorylated (Ser473) Akt and phosphorylated (Tyr416) Src expression. (B) WB analysis, as compared to control cells, CLDN1-overexpressed cells showed a strong increase of β-catenin, phosphorylated (Ser473) Akt and phosphorylated (Tyr416) Src expression.

By immunoblotting assay, we revealed that the amount of membrane β-catenin in suspended CLDN1-KD cells was decreased which might be responsible for the deficiency of cell-cell adhesion (Figure 8A). As Src and Akt signal pathways were involved in the cell-cell contact related anoikis resistance, both total and active (phosphorylated) Src and Akt protein expression were also tested. A significant decrease of the phosphorylated (Ser473) Akt and phosphorylated (Tyr416) Src could be detected in CLDN1-KD cells (Fig. 8A), reﬂecting a reduction of Akt and Src activity after detachment. Conversely, the expression of membrane β-catenin and the activities of phosphorylated (Ser473) Akt and phosphorylated (Tyr416) Src markedly increased in CLDN1-overexpressed cells than those in control cells (Fig. 8B).

### Overexpression of β-catenin in CLDN1-KD cell restores cell aggregation and anoikis resistance

**Figure 9:**
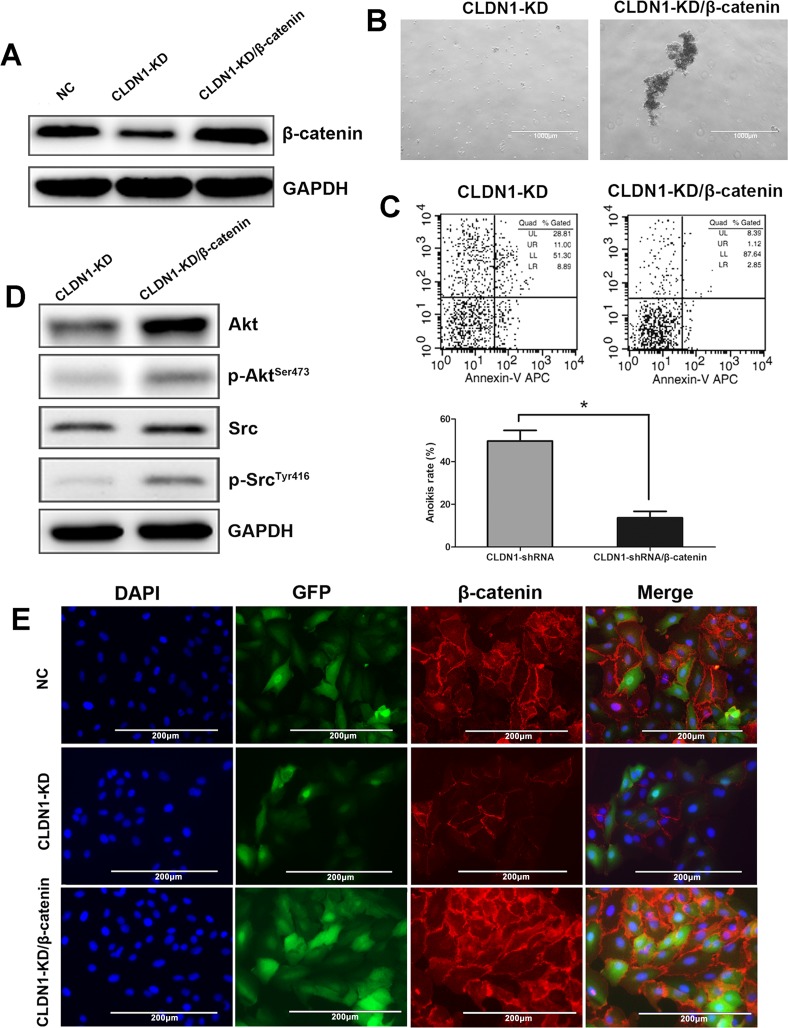
Overexpression of β-catenin in CLDN1-KD cell of HS-746T restored cell aggregation, anoikis resistance and survival signals of Akt and Src activation (A) WB analysis, the expression of β-catenin decreased in CLDN1-KD cells and re-increased in HS-746T/CLDN1-KD/β-catenin cells. (B) Representative photographs of cell aggregation after 6 hrs of suspension (original magnifications: ×40). (C) FCM analysis of cell apoptosis after 3h of suspension. Relative apoptotic rate of HS-746T/CLDN1-KD/β-catenin significantly decreased after 3h of suspension as compared to that of HS-746T/CLDN1-KD (*, P < 0.05). (D) WB analysis, protein expression of AKT, phosphorylated Akt, Src and phosphorylated Src significantly increased in HS-746T/CLDN1-KD/β-catenin as compared to those in HS-746T/CLDN1-KD. (E) Immunofluorescence staining, β-catenin localized particularly on membrane, and its expression decreased in CLDN1-KD cells and re-increased in HS-746T/CLDN1-KD/β-catenin cells.

To further explore the role of β-catenin in the CLDN1 regulated anoikis in gastric cancer, we transfected β-catenin into HS-746T/CLDN1-KD cells using lentiviral vector. As shown in Figure 9A and 9E, β-catenin expression, particularly on membrane, was restored when HS-746T/CLDN1-KD cells were transfected with β-catenin construct.

If β-catenin mediated the CLDN1 related anti-anoikis, re-expresiion of β-catenin in CLDN1-KD cells should restore the aggregation formation in suspension culture. Indeed, HS-746T/CLDN1-KD/β-catenin cells reformed large aggregations in suspension culture, but HS-746T/CLDN1-KD cells still lacked the ability to form aggregations (Figure 9B). The FACS analysis further showed decreased apoptotic rate in HS-746T/CLDN1-KD/β-catenin cells (Figure 9C), suggesting less anoikis in HS-746T/CLDN1-KD/β-catenin cells. In addition, overexpression of β-catenin also reactivated Akt and Src (Figure 9D). These data suggested that β-catenin may play a key role in the CLDN1 regulated anti-anoikis in gastric cancer cell.

## DISCUSSION

CLDN1, a member of tight junction proteins, maintains the integrity of the barrier function in normal epithelial cells [30]. Because CLDN1-induced acquisition of the malignant EMT phenotype, it was exploited as a biomarker for metastasis in liver cancer [21, 31, 32]. Increased CLDN1 protein level was related to metastasis capacity of colorectal cancer [33, 34]. CLDN1 was over-expressed in gastric cancer tissues, its expression was correlated with tumor invasiveness and metastasis, and was a prognostic factor of poor outcome [15, 17, 19, 35, 36]. However, CLDN1 regulated invasiveness is still unknown.

By using CLDN1-KD gastric cancer cell lines as a model, we found that CLDN1-KD cells exhibited decreased cell migration and invasion *in vitro*. Our findings are consistent with previous studies, suggesting an oncogenic role of CLDN1 [21, 27, 37]. Interestingly, we also found that the CLDN1-KD only impaired the gastric cancer cell growth in 3D culture, but not in monolayer culture. We further showed that CLDN1-KD significantly inhibited tumorigenesis and metastasis *in vivo*. As 3D culture is an *in vitro* experiment reflecting the anti-anoikis ability of cancer cells, and anti-anoikis ability is related to the tumorigenesis which has been confirmed in this study, we proposed that CLDN1 had an anti-anoikis potential in gastric cancer. Using anoikis experiment model, we found that CLDN1-KD cells were difficult to maintain cell-cell adhesion and form aggregate in suspended state, while control cells effectively formed aggregates. Meanwhile, cell apoptotic analysis showed that apoptosis was rapidly induced in CLDN1-KD cells. After establish other two gastric cancer cell lines with CLDN1 overexpression, we obtained opposite results to the CLDN1-KD cells. Overexpress of CLDN1 up-regulated cell migration, invasion and colony formation abilities, promoted cell aggregation and increased apoptosis of cells in suspension. Thus we considered that TJ protein CLDN1 is an anti-anoikis protein in gastric cancer and deficiency of CLDN1 expression suppressed cell aggregation and cell survival when deprived of cell-matrix adhesion.

Anoikis is a programmed cell death activated when cells are detached. For cancer cells, anoikis resistance is considered as a molecular prerequisite for the aggressive metastatic spread [26]. Anoikis has been described in several cell types, although it appears that cells of different tissue origin activate dissimilar pathways leading to anoikis [38]. Our studies suggest that CLDN1 may regulate anti-anoikis through different pathways. Firstly, EMT is an essential process for the acquisition of anoikis resistance. CLDN1 promotes EMT in hepatocellular carcinoma [32]. CLDN1 directly regulated cell transformation and interfered cell behavior and survival in colon cancer [27]. CLDN1 also showed an anti-apoptotic effect in different types of epithelial cancer cells and up-regulated cell resistance to the chemotherapy drugs [39, 40]. Other genes, such as Snail, Twist, HGF-R/cMet and NF-κB, are initiated and play a crucial role to evade anoikis by activating speciﬁc pro-survival signals [41].

Secondly, CLDN1 is critical in maintain cell-cell contact in suspended condition. Increasing evidence highlight that cell-cell adhesion supports cell survival. Adhesion junction and tight junction proteins may be involved in the mediation of cell-cell contacts. In the present study, we showed that knockdown or overexpress of CLDN1 induced variation of membrane β-catenin in gastric cancer cells. β-catenin is an important regulator of cell-cell adhesion. CLDN1 expression inhibited E-cadherin expression and activated β-catenin expression in colon cancer [27, 42]. β-catenin functioned as an oncogene by promoting the G1 to S phase transition and protecting cells from anoikis [43]. Hofmann C et al [28] reported that integrity of cell-cell contact could help maintaining survival signals of β-catenin, Src and PI3K/Akt, and prevent anoikis. We previously showed that the expression of CLDN1 was correlated with β-catenin expression in gastric cancer tissues, and CLDN1 expression could be regulated by β-catenin [17]. Here we further detected that membrane β-catenin could also be regulated by CLDN1, and CLDN1 regulated β-catenin expression may be a key pathway in the process of gastric cancer cell anoikis. Re-expression of β-catenin rescued anoikis caused by CLDN1-KD further strengthened our hypothesis that CLDN1 regulated anti-anoikis through mediating membrane β-catenin expression. Meanwhile, signal pathways of Akt (Ser475) and Src (Tyr416) were also activated. β-catenin mediated cell-cell adhesion might further affect the activation of survival signals such as Akt and Src. This potential link needs further study.

Taken together, our ﬁndings predict that the TJ protein CLDN1 may have a notable impact on the tumorigenic and metastatic capacities of human gastric cancer through maintenance of cell anoikis resistance. CLDN1 mediating anoikis is dependant on membrane β-catenin regulated cell-cell adhesion and survival signals of Akt and Src.

## MATERIALS AND METHODS

### Cell lines and Culture

Gastric cancer cell lines AGS (ATCC: CRL-1739), SNU-1 (ATCC: CRL-5971), HS-746T (ATCC: HTB-135), KATOIII (ATCC: HTB-103) and NCI-N87 (ATCC: CRL-5822), were obtained from American Type Culture Collection (Manassas, VA, USA). Other gastric cancer cell lines MKN-45, MKN-28, BGC-823 and SGC-7901 and immortalized human gastric epithelial cell line GES-1 were preserved in our institute. All cell lines were grown in Dulbecco's modified Eagle's medium (DMEM) supplemented with 10% fetal bovine serum (FBS), 80 U/ml penicillin, and 100 mg/ml streptomycin.

### Lentivirus-based CLDN1 knockdown and overexpress cells constructions

The short hairpin RNA (shRNA) lentiviral transduction particles for the CLDN1 knockdown experiment was obtained from Genepharma (Shanghai, CHINA). One CLDN1-specific shRNA construct ( shRNA sequence targeting CLDN1: GCCACAGCATGGTATGGCAAT ) and one “non-target” construct ( shRNA sequence: ACTACCGTTGTTATAGGTG) were transduced separately into BGC-823 and HS-746T cells. CLDN1 knockdown (CLDN1-KD) cells (designated here as BGC-823/CDLN1-KD and HS-746T/CLDN1-KD). The lentiviral transduction supernatant for CLDN1 overexpression was obtained from Genepharma (Shanghai, CHINA). The overexpression CLDN1 full-length cDNA was using primers 5 - ATCTCGAGATATGGCCAACGCGGGGCTG- 3 (F), 5 - GTGGATCCCTCCCACGTAGTCTTTCCCGCTG- 3(R) and subcloned into LV5-EF1a-GFP vector to generate the LV5-EF1a-GFP/CLDN1 construct. The AGS and NCI-N87 cells were transfected by LV5-EF1a-GFP/CLDN1 for CLDN1 overexpression and the vector of LV5-EF1a-GFP was used as control. Another construct of LV5-EF1a-GFP/β-catenin lentiviral transduction particles for ectopic overexpression of β-catenin was also purchased from Genepharma (Shanghai, CHINA) and transfected into HS-746T/CLDN1-KD cells. Stable transfected cell clones was selected with puromycin and screened by quantitative reverse transcription-polymerase chain reaction (QRT-PCR), Western blot analysis and immunofluorescence detection.

### Quantitative RT-PCR (QRT-PCR)

Total RNA was isolated from cultured cells using the RNeasy mini kit (Qiagen, GER) and cDNA was synthesized with oligo (dT) primers by using of a SuperScript first-strand cDNA synthesis kit (Invitrogen, USA) according to the manufacturer's protocols. Gene expression was assessed by qRT-PCR using an Applied Biosystems 7500 Fast Sequence Detection System (Life Technologies Corporation, CA, USA). The transcript of the housekeeping gene, Glyceraldehyde-3-phosphate dehydrogenase (GAPDH) gene was used as endogenous control to normalize expression data. Primers used for qRT-PCR analysis of CLDN1 expression are 5′- GCCCTGCCCCAGTGGAGGAT -3′ (forward) and 5′- CGGGTTGCTTGCAATGTGCTGCT -3′ (reverse). Primers used for analysis of GAPDH are 5′-TTGGCATCGTTGAGGGTCT-3′ (forward) and 5′-CAGTGGGAACACGGAAAGC -3′ (reverse). The comparative Ct (threshold cycle) method was used to calculate the relative changes in gene expression.

### Western Blotting and Immunofluorescence Staining

Western blotting and immunofluorescence staining were done according to our standard protocol, as described previously [17].

### Cell Cycle and Apoptosis Analysis By Flow Cytometry

For cell cycle analysis, the cells were ﬁxed in ice-cold 70% ethanol and stored at 4°C overnight. Fixed cells were washed with PBS, treated with 100 μg/ml RNase A at 37°C for 20 min. After staining with propidium iodide (50 μg/ml), the cells were subjected to fluorescence activated cell sorting (FACS) on a FACScan (Beckman Instruments, USA). The cell debris and ﬁxation artifacts were gated out and the cell populations that were at the G0/G1, S and G2/M phases were quantiﬁed using the Modﬁt software.

For apoptosis analysis, an Annexin V-FITC Apoptosis Detection Kit I (BD Pharmingen, USA) was used according to the manufacturer's instructions. Brieﬂy, cells were washed with PBS and resuspend in 1× Binding Buffer, then, 5 μl FITC Annexin V and 5 μl PI were added to 100 μl of the cell suspension and incubated for 15 min in the dark. After incubation, 400 μl 1× Binding Buffer was added. The apoptosis was analysed by FACScan using the Cell-Quest software. Annexin V-FITC-positive and PI-negative cells were undergoing apoptosis.

### Cell Proliferation Assays

Cell proliferation was assessed by the colorimetric water-soluble tetrazolium salt (WST) assay using a Cell Counting Kit-8 (Dojindo, Kumamoto, Japan) according to the manufacturer's instructions. The negative control cells (BGC-823/NC and HS-746T/NC) and stably CLDN1-KD cells (BGC-823/CLDN1-KD and HS-746T/CLDN1-KD) (1×10^3^ ) were seeded into 96-well plates in triplicate, and cell proliferation was documented every 24 h for 4 days. The number of viable cells was assessed by measurement of the absorbance at 450 nm using a Saﬁre2 microplate reader (Tecan, Swiss).

### Plate Colony Formation Assay and Soft Agar Colony Formation Assay

Plate colony formation (PCF) assay was done using six-well plates. Cells (1 × 10^3^) were seeded into each well with 2 ml DMEM supplemented with 10% FBS. Culture medium was changed every 3 days. After 14 days, the resulting colonies were fixed with methanol at –20°C for 5 min, and then stained with crystal violet. Only clearly visible colonies (diameter > 50 μm) were counted.

For soft agar colony formation (SACF) assay, cell suspension was mixed with 0.3% soft agar in DMEM containing 10% FBS and layered in triplicate onto 0.6% solidiﬁed agar in DMEM containing 10% FBS (1 × 10^3^ cells/well). After 14 days culture, colonies containing 50 cells or more were counted under a microscope at ×100 magnification as previously described.

### *In Vitro* Cell Migration and Invasion Assay

Cells were cultured to about 80% confluence and serum starved overnight. Transwell filters (BD Biosciences, USA) of 8-μm pore size were coated overnight with 100 μl of 1:6 diluted Matrigel (BD Biosciences, USA). 5 × 10^4^ cells after overnight starvation were plated in the coated filters in 100 μl of serum-free medium. Five hundred microliters of the same medium containing 10% FBS was placed in the lower chamber to act as a chemoattractant. After 24 hours of incubation, cells that had invaded to he lower surface of the collagen-coated membrane were fixed with cold methanol for 10 minutes, then stained with 0.01% crystal violet in 20% ethanol, and counted in 5 randomly selected fields under a light microscope. The experiment was performed twice with each sample in triplicate. To assess cell migration, assays were carried out as above except that cells were plated on top of uncoated (Matrigel-free) filters.

### *In Vivo* Tumorigenicity and Metastasis Assay

Male BALB/c nu/nu mice at age 4 to 5 weeks, purchased from Institute of Zoology Chinese Academy of Sciences, were housed in the Animal Laboratory Unit, Shanghai Jiao Tong University School of Medicine, China. All experiments were performed in accordance with the official recommendations of the Chinese animal community. Five mice were included in each group in all experiments, and each experiment was performed twice. For tumor xenograft model, the cells (1 × 10^6^) were inoculated subcutaneously into the right flank regions. Tumor volume was checked every 3 days calculated using the formula: V =4/3π × L/2 × (W/2)2, where L is the mid-axis length, and W is the mid-axis width, and plotted as a function of time to generate the *in vivo* growth curves. Animals were sacrificed four weeks after cell inoculation. Tumor specimens were collected, fixed in formalin, and embedded in paraffin, and subjected to HE staining. In pulmonary metastasis study, 100 μl (1 × 10^6^ cells) suspension per mouse was injected into caudal vena. Mice were sacrificed by cervical decapitation after eight weeks of caudal vena injection, and the lungs with metastatic masses were removed and ﬁxed with 10% buffered formalin for further morphological analysis.

### Anoikis Analysis

Anoikis was induced by plating cells (2×10^6^) on poly-HEMA (Sigma) coated 30 mm culture dishes and shaking at 80 rpm at 37°C in a 5% CO_2_ humidified incubator. At 1h, 3h and 6h after plating, the cells were photographed and collected to examine. The apoptosis was analyzed by ﬂow cytometry. Apoptosis-related proteins such as cleaved caspase-3 (CST, USA) and cleaved PARP (CST, USA) were detected by Western blotting.

### TUNEL Analysis

TUNEL assay was performed on paraffin-embedded tissue sections using the one-step TUNEL apoptosis assay kit (Beyotime, Shanghai, China) according to the manufacturer's instructions. After dewaxing and proteinase K treatment, samples were incubated with TUNEL reaction mixture for 1 h at 37°C in the dark and then washed twice in PBS. The condensed or fragmented nuclei of apoptotic cells were observed using ﬂuorescence microscopy at × 200 magnification.

### Statistical Analysis

Statistical analyses were performed using SPSS 13.0 software package (School of Medicine, Shanghai Jiao Tong University). The distributions of quantitative variables were tested. Normally and non-normally distributed quantitative variables are presented as means ± standard deviation. Continuous variables were compared between groups using an unpaired t-test and a paired t-test within each group. Categorical variables were compared using Pearson χ2 test. For small samples, analysis of variance and Fisher's exact test were used to analyze continuous and categorical variables, as appropriate. P < 0.05 was selected as the statistically significant value.

## SUPPLEMENTARY MATERIALS FIGURES


