# Supplementary material for: Claudin-1 enhances tumor proliferation and metastasis by regulating cell anoikis in gastric cancer
Source: Oncotarget. 2014 Dec 2;6(3):1652–65. doi: 10.18632/oncotarget.2936 (PMC4359322; doi:10.18632/oncotarget.2936)
Supplement: Supplementary file 1 [file oncotarget-06-1652-s001.pdf]

Claudin-1 enhances tumor proliferation and metastasis by regulating cell anoikis in gastric cancer

Supplementary Material

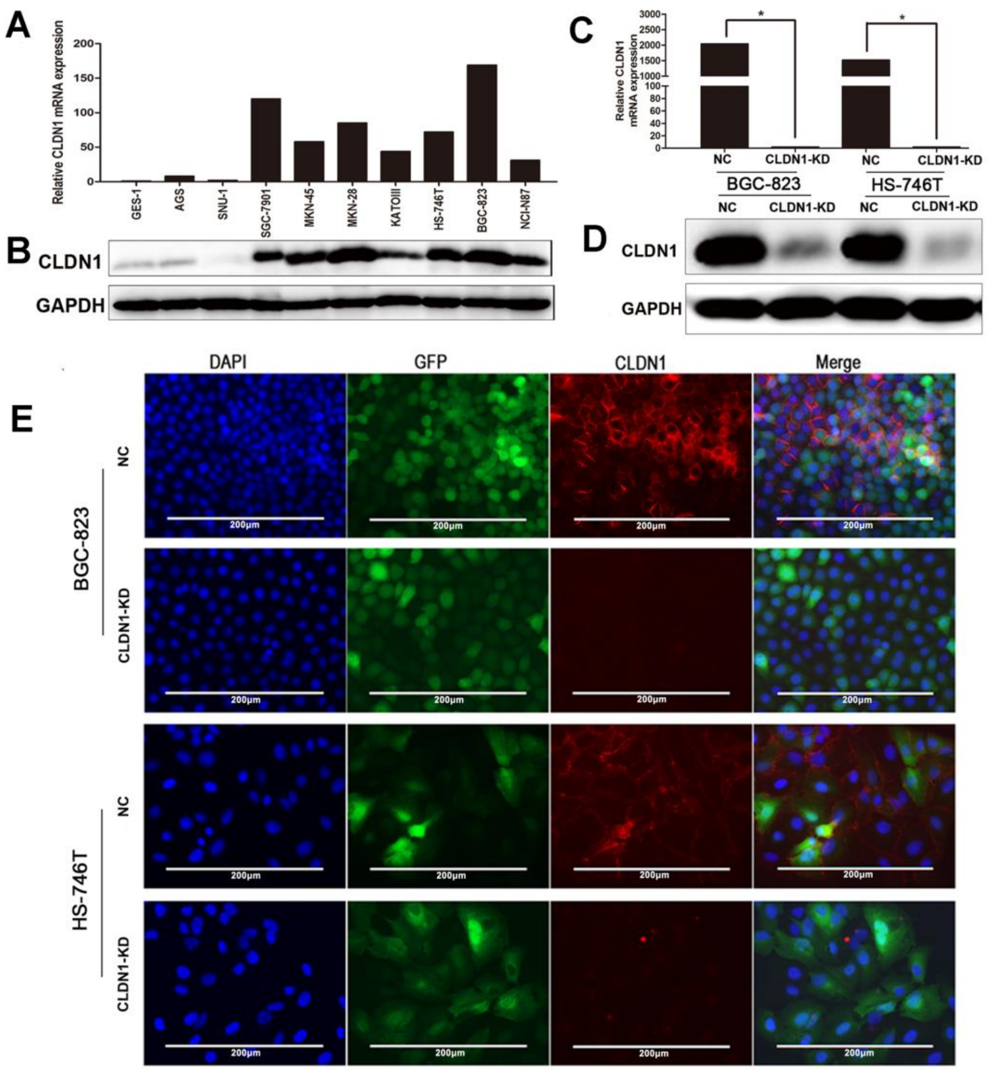

Figure S1: CLDN1 expression in gastric cancer lines and effects of CLDN1-KD in gastric cells of BGC-823 and HS-746T. (A) mRNA levels of CLDN1 in immortalised gastric epithelial cell line GES-1 and 9 gastric cancer cell lines were examined by using qRT-PCR. (B) Protein expression of CLDN1 in immortalised gastric epithelial cell line GES-1 and 9 gastric cancer cell lines was examined by using immunoblotting. (C) mRNA levels of CLDN1 in cells of BGC-823/CLDN1-KD and HS-746T/CLDN1-KD and control cells of BGC-823/NC and HS-746T/NC were examined by using qRT-PCR. (D) Protein expression of CLDN1 in cells of BGC-823/CLDN1-KD and HS-746T/CLDN1-KD and control cells of BGC-823/NC and HS-746T/NC were examined by using immunoblotting. Cell line GES-1 was used as a control. GAPDH was used as loading control. (E) Immunofluorescence staining of CLDN1 in cell line BGC-823 and HS-746T with knockdown of CLDN1 (original magnifications: ×200). CLDN1 (red) expression was significant decreased in BGC-823/CLDN1-KD and HS-746T/CLDN1-KD as compared to negative control cell BGC-823/NC and HS-746T/NC, respectively.

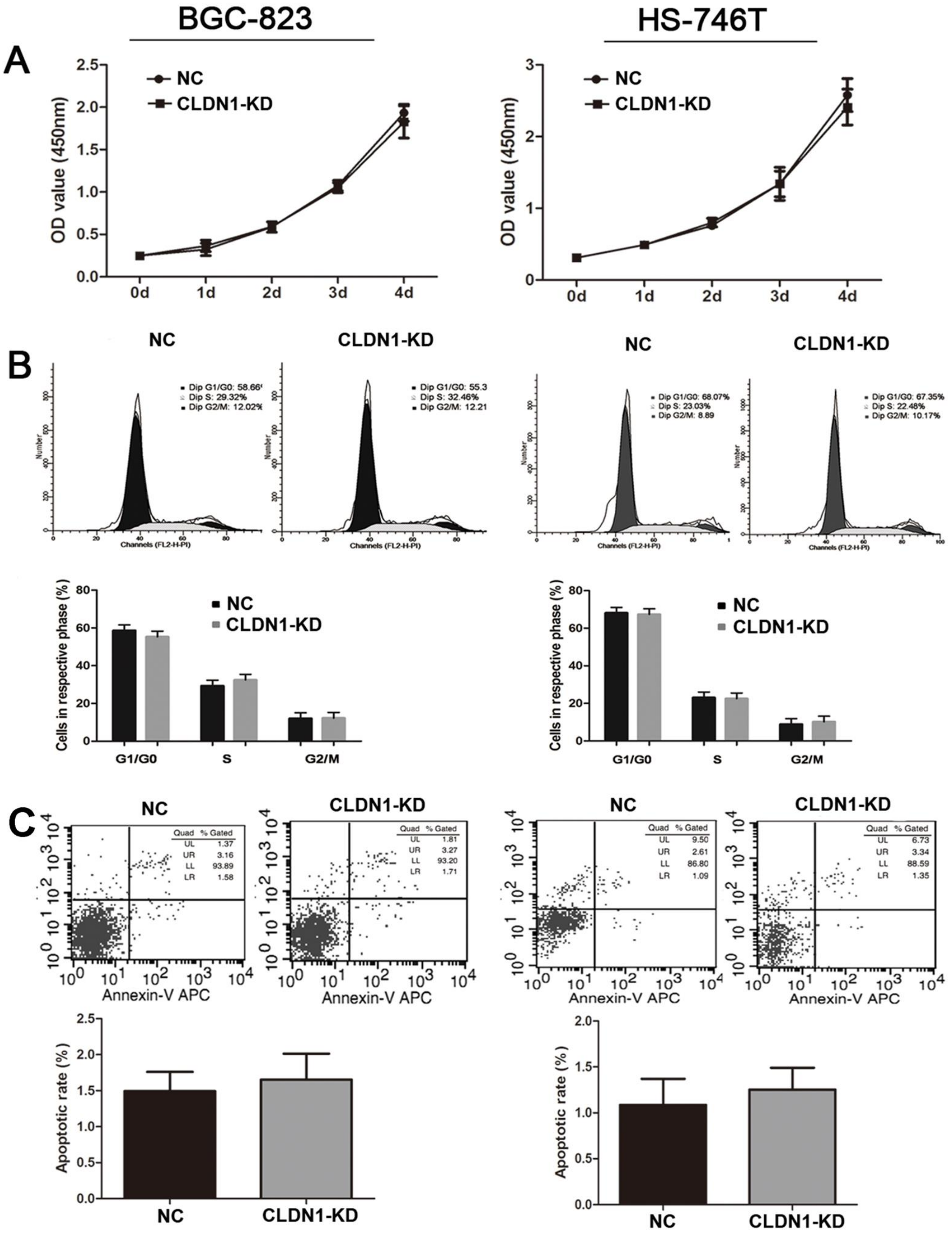

Figure S2: Knockdown of CLDN1 didn't affect cell growth, cycle and apoptosis in monoplayer culture. (A) Monolayer growth rates of cells were determined by WST assay. (B) FCM analysis of cell cycle distribution. (C) FCM analysis of cell apoptosis and cell apoptotic distribution. Values represent the mean ± SD from at least three separate experiments, each conducted in triplicate.

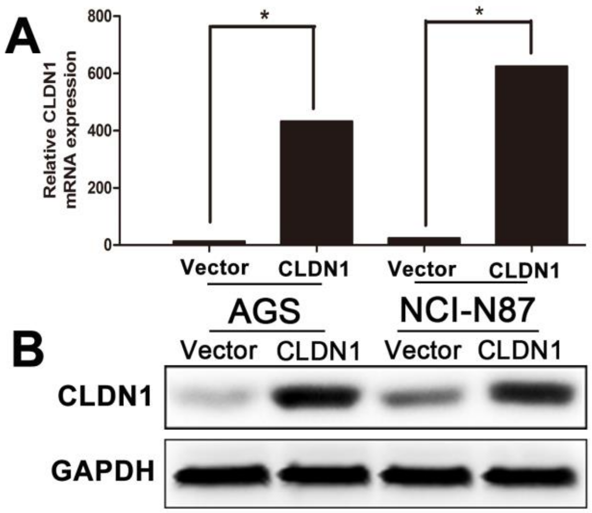

Figure S3: Overexpression of CLDN1 in gastric cell lines of AGS and NCI-N87. (A) mRNA levels of CLDN1 in cells of AGS/CLDN1 and NCI-N87/CLDN1 and control cells of AGS/Vector and NCI-N87/Vector were examined by using qRT-PCR. (D) Protein expression of CLDN1 in cells of AGS/CLDN1 and NCI-N87/CLDN1 and control cells of AGS/Vector and NCI-N87/Vector were examined by using immunoblotting. GAPDH was used as loading control.
